# Examining DNA Breathing with pyDNA-EPBD

**DOI:** 10.1101/2023.09.09.557010

**Published:** 2023-09-12

**Authors:** Anowarul Kabir, Manish Bhattarai, Kim Ø. Rasmussen, Amarda Shehu, Anny Usheva, Alan R Bishop, Boian S Alexandrov

**Affiliations:** 1 Theoretical Division, Los Alamos National Laboratory, Los Alamos, 87544, NM, 87102.; 2 George Mason University, 4400 University Dr, Fairfax, VA 22030.; 4 Brown University, 69 Brown St Box 1822, Providence, RI 02912.

**Keywords:** DNA breathing, DNA bubbles, DNA non-linear dynamics, EPBD

## Abstract

**Motivation:**

The two strands of the DNA double helix locally and spontaneously separate and recombine in living cells due to the inherent thermal DNA motion.This dynamics results in transient openings in the double helix and is referred to as “DNA breathing” or “DNA bubbles.” The propensity to form local transient openings is important in a wide range of biological processes, such as transcription, replication, and transcription factors binding. However, the modeling and computer simulation of these phenomena, have remained a challenge due to the complex interplay of numerous factors, such as, temperature, salt content, DNA sequence, hydrogen bonding, base stacking, and others.

**Results:**

We present pyDNA-EPBD, a parallel software implementation of the Extended Peyrard-Bishop- Dauxois (EPBD) nonlinear DNA model that allows us to describe some features of DNA dynamics in detail. The pyDNA-EPBD generates genomic scale profiles of average base-pair openings, base flipping probability, DNA bubble probability, and calculations of the characteristically dynamic length indicating the number of base pairs statistically significantly affected by a single point mutation using the Markov Chain Monte Carlo (MCMC) algorithm.

## 1 Introduction

**Fig. 1:**
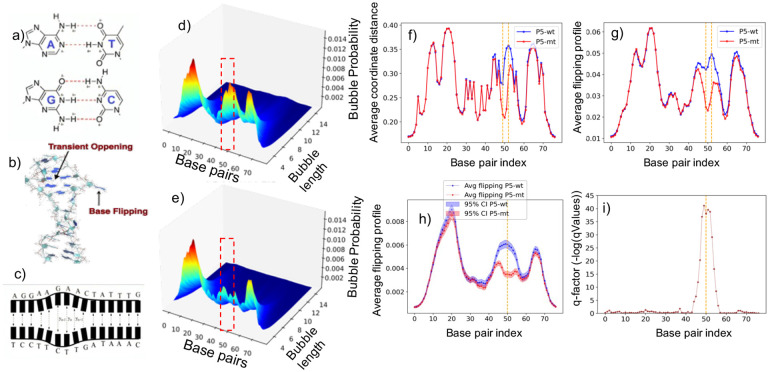
DNA Breathing Dynamics and Analysis. (a) The primary governance of macromolecules through hydrogen bonds (H-bonds). (b) Representation of a single base “flipping out of the stack,” showcasing a phenomenon known as DNA breathing. (c) Illustration of consecutive base-pairs breaking the H-bonds and opening simultaneously, referred to as DNA bubbles. (d-e) 3D surface plots highlighting the change in bubble intensity across varied lengths and base pairs (bps) for threshold value 1.5 under two conditions: P5 wild (d) and P5 mutant (e). (f-g) Average Coordinates profiles for AAV P5 wild (f) and mutant-promoter (g) sequences at individual base pairs, with the orange vertical block indicating nucleotide substitutions from AG to TC at the 50 and 51 positions (zero-indexed). (h) Average flipping profiles alongside the (i) corresponding q-factor for AAV P5 wild and mutant-sequence. For all the experiments, we set a minimum of 100 MCMC simulations using various initial conditions, setting the temperature to 310 Kelvin and employing 50,000 preheating steps followed by 80,000 post-preheating steps.

The structural integrity of biological macromolecules is primarily governed by hydrogen bonds (H-bonds) Figure 1a), which have natural vibration frequencies in the terahertz range Turton et al. (2014). H-bonds are much weaker (~ few *k*_*B*_Ts) than covalent bonds, causing the macromolecules to experience slow conformational motion resulting from the inherent thermal fluctuations at physiological temperatures.

In living cells, the thermal fluctuations spontaneously induce local opening and closing of the DNA helix, referred to as DNA breathing, which can occur as a single base flipping out of the stack (base flipping) Figure 1b), or as a few consecutive base-pairs breaking the H-bonds and opening simultaneously (DNA bubbles) Figure 1c). Large amplitude bubbles describe local regions of DNA melting (denaturation).

Single base-pair opening/flipping, has been studied by tracking the exchange of protons from imino groups with water Guéron et al. (1987). DNA breathing is essential for DNA transcription, transcription factor (TF) binding, replication, enzymatic repair, and base pair methylation, Kli-mašauskas and Roberts (1995); Roberts and Cheng (1998); Choi et al. (2004); Dellarole et al. (2010); Fenwick et al. (2011); Alexandrov et al. (2012); Phelps et al. (2013),It was also observed that single base pair genetic variants, e.g., in the flanks of TF motifs) may influence DNA breathing dynamics and TF binding Alexandrov et al. (2011); Jablensky et al. (2012); Alexandrov et al. (2012); Duan et al. (2014).

The thermo-mechanical characteristics of DNA have been precisely described using a variety of models. Here We employ the mesoscopic Extended-Peyrard-Bishop-Dauxois (EPBD) model to examine DNA breathing dynamics. EPBD is an expansion of the Peyrard-Bishop-Dauxois nonlinear model of DNA and includes sequence-specific base-pair stacking potentials DNA breathing dynamics. The probabilities for local DNA openings obtained from the EPBD model are equilibrium properties of the underlying free energy landscape, and similar information can be obtained from various thermodynamical models, such as the Poland-Sheraga model.The parameters of the EPBD model have been derived from DNA melting experiments Ares et al. (2005); Alexandrov et al. (2009). The EPBD derived trajectories contain unique information about the relative lifetimes of the DNA bubbles which cannot be obtained by thermodynamical calculations. Another advantage of EPBD is its single nucleotide resolution.

By using EPBD simulations to locate DNA breathing hotspots, we were able to:(i) design (computationally) single-point mutations that silence breathing dynamics of transcription start sites (TSS), and to demonstrate (experimentally) that such mutations mutations also alter transcription, without affecting the TF binding Alexandrov et al. (2010); ii) design (computationally) mismatches, which enhance bubble formation, that can lead (experimentally) to bidirectional transcription initiation in the absence of basal TFs Alexandrov et al. (2009); (iii) design (computationally) base-pair substitutions or methylations, that change local DNA breathing and show (experimentally) that TF-DNA binding changes accordingly Nowak-Lovato et al. (2013). Further, EPBD simulations have indicated significant changes in the spatio-temporal characteristics of double-stranded DNA in the presence of a UV-dimer Blagoev et al. (2006) (which can be interpreted as an effective increase of the local temperature).

The EPBD modeling framework has been used to simulate the influence of non-ionizing terahertz (THz) radiation on DNA breathing and demonstrated Alexandrov et al. (2010) that, at sufficient exposure, DNA bubbles can appear through a nonlinear resonance mechanism. Such resonance bubble formations may have a direct effect on transcription, replication, and DNA-protein binding, thus providing/suggesting a connection between THz radiation and biological function. Experimentally, it have been demonstrated that intense ultra-short THz pulses can indeed lead to non-thermal effects, including, modified gene expression profiles, gene-specific activation/repression, and changes in stem-cell differentiation Bock et al. (2010); Alexandrov et al. (2011, 2013); Titova et al. (2013); Perera et al. (2019); Shang et al. (2021). Importantly, based on the optical Kerr effect, it was shown that the interstrand H-bond modes (bubbles) of DNAare coherent delocalized/non-linear phonon modes in the THz range at physiological conditions González-Jiménez et al. (2016).

Experimental studies of looping of ultra-short DNA sequences revealed a discrepancy of up to six orders of magnitude between experimentally measured and theoretically predicted Jacobson-Stockmayer’s J-factors Vafabakhsh and Ha (2012); Alexandrov et al. (2016), suggesting that, in addition to the elastic moduli of DNA, the presence of local single-stranded ”flexible hinges” in DNA (i.e., DNA bubbles) can assist the loop formation Yan and Marko (2004). Combining the Czapla-Swigon-Olson structural model of DNA with the EPBD model Alexandrov et al. (2017), (without changing any of the parameters of the two models), it was shown that the calculated J-factors of calculated J-factors of ultra-short DNA sequences are within an order of magnitude of the experimental measurements

## 2 Material and Methods

Our pyDNA-EPBD model utilizes Markov chain Monte Carlo (MCMC) protocol. The specific algorithm is summarized in Figure 1l). We use the standard Metropolis algorithm to produce an equilibrium state of the system: First, a base pair, *n*_0_, is selected at random, then a strand (left or right) is selected randomly and a new value of the variable $u_{n_{0}}$ or $v_{n_{0}}$ is proposed according to a thermal (at the given temperature) Gaussian distribution at this base pair. The proposed value is accepted according to the Metropolis probability, *P*: (a) *P* = 1 if the energy, *E*_*new*_, of the new configuration is lower than the energy, *E*_*old*_, of the old configuration; and, (b) *P* = exp[(*E*_*old*_ − *E*_*new*_)/*k*_*B*_*T*], if *E*_*new*_ > *E*_*old*_. The process is continued after thermal equilibrium is reached in the measurement phase. For each temperature, we performed several simulations (runs). In each of these runs, we compute the transfers opening profile *y*_*n*_. Subsequently, the displacements *y*_*n*_ of each base pair is recorded at every Δ*t* (intervals of selected MCMC steps). By conducting numerous runs, each with different initial conditions, we derived the average displacement/opening profile, denoted as 〈*y*_*n*_〉.

One of the main characteristics of DNA breathing and DNA bubbles is the displacement, *y*_*n*_, (see Fig 1e) from equilibrium position for each base pair in the sequence. This displacement signifies the transverse stretching of the Hbonds between the complementary nucleotides. An advantage of the *y*_*n*_ profile is that it eliminates the need for window averaging typically required in thermodynamic calculations, thus making the average displacement/opening profiles sensitive to single base pair substitutions. The *y*_*n*_ profile can be efficiently calculated by MCMC simulations, yielding results equivalent to those obtained by averaging over Langevin dynamics trajectories Choi (2004). Below we introduce the main DNA breathing characteristics that our tool pyDNA-EPBD calculates:

- **Average Coordinates:** Refers to the averaged transverse displacements, 〈*y*_*n*_〉, i.e. the displacements, *y*_*n*_, averaged over the thermal fluctuations. The displacement profile, 〈*y*_*n*_〉, is one of the distinct properties of the DNA breathing characteristics, which quantifies the extent to which each of the base pairs of the DNA sequence is “open” in equilibrium, i.e., the extent to which the H-bonds between the bps are stretched due to the thermal fluctuations.
- **Base Flipping Probability:** Describes a particular kind of base movement in which at least one of the bases, in a base pair, flips out of the stack. The flipping exposes the base to the surrounding environment, which can be important for a variety of biological processes such as DNA repair, replication, and TF binding. The propensity of flipping characterizes this transition, by determining the fraction of disrupted Hbonds (openings) between complimentary nucleotides, i.e., the fraction of bps for which *y*_*n*_ exceeds a certain threshold distance, as a function of temperature.
- **DNA Bubble Probability:** Refers to extended DNA regions where the double helix unwinds and the two strands temporarily separate, due to the thermal motion. These transient opening or denaturation bubbles are part of the natural dynamics of DNA and can play a role in processes such as the initiation of transcription, replication and TF binding propensity. The probability for bubble existence, of a bubble begining at bp *n*-th, of length *l* bps and with a displacement *y*_*n*_ exceeding a given threshold (*thr*) (in Å) is a three dimensional (3D) tensor, *P*(*n, l,thr*), which can be computed as, Ref. Alexandrov et al. (2006),
(1) $$P(n,l,thr)={\langle \frac{1}{t_{s}}\sum_{k=1}^{k_{\text{max}}}\Delta t(k,n,l,thr)\rangle }_{M},$$
  Here, 〈·〉_*M*_ denotes averaging over M runs, *t*_*s*_ is the total number of of MCMC simulation steps. The bubble duration Δ*t* (in MCMC steps) is a nonlinear function of its initial position (the beginning of the bubble), *n*, on its length, *l*, and amplitude threshold, *thr*.
- **Dynamic Length:** Since the average flipping profile represents the breathing propensity of each of the base pairs in a DNA region, we need a way to compare breathing profiles of two alleles that differ, e.g., due one or two SNPs. The dynamic length quantifies the statistical significance of the differences between two such flipping profiles. Specifically, the dynamic length, quantifies how many and precisely which base-pairs experience statistically significant changes caused by the introduced SNPs. We quantified the dynamic length by the concept of a “q-factor” as a function of the position of the base pair, which has been used to estimates the false discovery rate (FDR) in multiple stochastic simulations/experiments Lai (2017).

The average coordinates profile, (Figure 1(f)), the flipping probability profile, (Figures 1(g)), the 3D bubble probability tensor (Figures 1(d–e)), and the dynamic length profiles (Figure 1(h–i)), are key factors for understanding the DNA breathing. The corresponding details are provided in the supplementary material.

## 3 Usage

The pyDNA-EPBD tool is distributed under the 3-Clause BSD License, and it is compatible with Windows, MacOS, and Linux-based systems. pyDNA-EPBD is designed to work seamlessly in large-scale deployments, whether on high-performance computing clusters or cloud infrastructures like Amazon Web Services. Users can provide the input DNA sequence in common formats, such as FASTA or text file. Upon running simulations with specific parameters, pyDNA-EPBD generates the following characteristic outputs: the average coordinates profile, the flipping probability profile, the 3D probability bubble tensor, and the dynamic length profile. The details on hyperparameters, experimental configuration for reproducibility and analysis is provided in the supplementary material.

## Supplementary Material

1

## Data Availability

pyDNA-EPBD is Open Source Software published under the 3-Clause BSD License and can be found at https://github.com/lanl/pyDNA_EPBD.
