# Supplementary material for: Examining DNA Breathing with pyDNA-EPBD
Source: bioRxiv. 2023 Sep 12:2023.09.09.557010. Preprint. [Version 1] doi: 10.1101/2023.09.09.557010 (PMC10515784; doi:10.1101/2023.09.09.557010)
Supplement: 1 [file NIHPP2023.09.09.557010V1-supplement-1.pdf]

bioRxiv preprint doi: https://doi.org/10.1101/2023.09.09.557010; this version posted September 12, 2023. The copyright holder for this preprint (which was not certified by peer review) is the author/funder. This article is a US Government work. It is not subject to copyright under 17 USC 105 and is also made available for use under a CC0 license.

Supplementary Materials

A Implementation

To study DNA breathing dynamics, we used the mesocopic EPBD model, which is an extension of the original Peyrard-Bishop-Dauxois model Dauxois et al. (1993) that includes sequence-specific stacking potentials. A comment on the choice of model is perhaps appropriate, as many models have been used to study the mechanical properties of DNA. Most of them are purely thermodynamical models parameterized on the basis of measurements of equilibrium thermodynamical properties. The probabilities for local DNA opening obtained from the EPBD model are also equilibrium properties of the underlying free energy landscape, and essentially the same information can be obtained from various available thermodynamical models, such as the Poland-Sheraga model Richard and Guttmann (2004). However, it should be noted that the EPBD model is a dynamical model that is strongly nonlinear and admits breathing solutions, which constitute transient but relatively long-lived openings of the double helix, that are interconnected with the local bending propensity. The EPBD derived trajectories contain the information about the lifetimes of the DNA transient openings (bubbles). This type of information cannot be obtained by purely thermodynamical calculations. Explicit accounting of the dependence on the solvent conditions such as salt, temperature, as well on the twist of the DNA, can lead to long-lived bubbles with enhanced lifetimes. Another advantage of the EPBD model is its single nucleotide resolution. With a thermodynamical model, the calculation of a property profile typically requires window averaging over 100–500 bps, which limits one's ability to distinguish the property profiles of two closely related sequences. In contrast, window averaging is not used in EPBD calculations, and as a result, the effects of even single bp changes can be readily determined.

The EPBD model is a quasi-two-dimensional nonlinear model that describes the transverse opening motion of the complementary strands of double-stranded DNA, while distinguishing the two sides (right-u_n and left-v_n) of the DNA double strand. The Hamiltonian potential surface V_{EPBD} of the EPBD model is defined by the summation of the Morse Potential (first part) and stacking energy term (second term) of the two neighboring bps at every bp of the input DNA fragment, see Eqn. 2.

$$V_{EPBD} = \sum_{n=1}^{N} U[u_n; v_n] + W[u_n; u_{n-1}; v_n; v_{n-1}] \quad (2)$$

The Morse potential incorporates the Hydrogen bonds connecting two bases belonging to opposite strands, the repulsive interactions of the phosphates, and the surrounding solvent effects. The parameters D and a in this term denote the nature of the n-th bp, i.e. A–T or G–C, see Eqn. 3.

$$U[u_n; v_n] = D_n(e^{-a_n(u_n - v_n)} - 1)^2 \quad (3)$$

The stacking energy potential is defined between two neighboring bps using their status variables y_n and y_{n-1} as in Eqn. 4 which is defined by the distance of the bp, $y_n = u_n - v_n$. The K term determines the coupling constants between the left and right two neighboring bases.

$$W[u_n; u_{n-1}; v_n; v_{n-1}] = \frac{K^u_{n;n-1}}{2}(u_n - u_{n-1})^2 \frac{K^v_{n;n-1}}{2}(v_n - v_{n-1})^2 + \frac{\rho}{4} e^{-\beta[(u_n - v_n)+(u_{n-1} - v_{n-1})]} \left(\sqrt{K^u_{n;n-1}}(u_n - u_{n-1}) - \sqrt{K^v_{n;n-1}}(v_n - v_{n-1})\right) \quad (4)$$

MCMC methods, for sampling probability distribution, constructs Markov chain with the target distribution. By recording states of the chain, one can obtain samples of a desired distribution. The more the number of samples, the more the samples distribution gets closer to the actual distribution. The MCMC protocol used in pyDNA-EPBD model is similar to what Ares *et. al* Ares et al. (2005) applied to understand the bubbles activity in DNA melting.

To calculate the average displacement or opening profile for a given DNA sequence at a specific temperature, we utilized the pyDNA-EPBD model in our MCMC simulations as detailed in previous literature, summarized in Algorithm 1 and in Figure 2. The standard Metropolis algorithm was applied to generate an equilibrium state in each simulation run. Subsequently, the displacements y_n of each base at selected time intervals were recorded. By conducting numerous simulation runs, each with different initial conditions, we derived the average displacement/opening profile, denoted as y_n, for the DNA sequence under investigation.

A single step in the simulation involves selecting a random base-pair to do random displacement at the left or right base. Based on the displacement occurred, new DNA state and corresponding energy is calculated using Morse-potential (Eqn. 3) and stacking energy (Eqn. 4) in the EPBD model (Eqn. 2). The new DNA state is updated if the energy tends to an equilibrium state or the energy change due to this random displacement satisfies a Metropolis criteria. The criteria is defined such that the energy change due to the random displacement follows a simulation temperature scaled log-uniform distribution.

Figure 3 shows an example distribution of the chosen Metropolis criteria at 310.0 Kelvin. 130,000 samples are randomly generated from the criteria for demonstration. The violin plot shows the box-and-whisker plot (red-ish box with

bioRxiv preprint doi: https://doi.org/10.1101/2023.09.09.557010; this version posted September 12, 2023. The copyright holder for this preprint (which was not certified by peer review) is the author/funder. This article is a US Government work. It is not subject to copyright under 17 USC 105 and is also made available for use under a CC0 license.

Algorithm 1: Metropolis-Hasting MCMC algorithm

```
Data: seq, temp, totalSteps
DNAState ← initDNAState(seq);
Energy ← initDNAEnergy();
for step = 1 to totalSteps do
    for i = 1 to len(seq) do
        nthBP ← random(1, length(seq));
        lor ← random();
        dx ← normalvariate(mu = 0.0, sigma = 1.0);
        newDNAState ← doRandomDisplacement(nthBP, lor, dx);
        newEngery ← computeTotalEnergy(newDNAState);
        if newEngery == Energy then
            return;
        else if newEnergy < Energy then
            DNAState ← newDNAState;
            Energy ← newEngery;
        else if Energy − newEngery satisfies Metropolis criteria then
            DNAState ← newDNAState;
            Energy ← newEngery;
    collectDNABreathingDynamics(step, DNAState, Energy)
```

vertical caps) along with the kernel density estimate (KDE) (blue area). The KDE shows the continuous probability density using a Gaussian kernel which is skewed towards zero, indicating that a new DNA state will be selected during the MCMC simulations if the energy change is small. This criteria ensures that the simulation does not get stuck in the local energy minima, instead, it will explore the energy landscape to reach a possible global minima. In particular, the box and whisker plot shows that 75% of the samples are within range from 0 to 51.78 Kelvin (up to third quartile) which gives the empirical evidence of such exploration. In essence, this Metropolis criteria ensures that the simulation reaches an equilibrium state, possibly a global minima, by examining enough Markov samples in the energy landscape.

B Results and Discussions

First, we provide the specifics of datasets and the simulation configurations used throughout the study. Subsequently, we discuss the DNA breathing dynamics, such as the coordinates of bps, the flipping of bases, the formation of bubbles, and the q-factor. We also detail an utility of the breathing characteristics for Transcription Factor (TF)-DNA binding specificity prediction. Finally, we analyze runtimes of our pyDNA-EPBD model as a function of the number of bps consisting each DNA sequence.

B.1 Datasets

This section briefly discusses the datasets utilized throughout the study, necessary to understand different utilities and perspectives of our pyDNA-EPBD model.

- **Adeno-associated virus (AAV) P5 promoter:** Experimental data suggest that a spontaneous double strand DNA (dsDNA) separation at the transcriptional start site is likely to be a requirement for transcription initiation in several promoters Alexandrov et al. (2009). To study this phenomena using our pyDNA-EBPD model, we utilize the strand separation (bubble) dynamics of 77-bp-long AAV P5 promoter (wild-type (wt) sequence: "GCGCGTGGCCATTTAGGGTATATATG-GCCGAGTGAGCGAGCAGGATCTC-CATTTTGACCGCGAAATTTGAACGGCGC"). We also study the control non-promoter sequence (77 bp) (mutant-type (mt) sequence: "GCGCGTGGCCATTTAGGGTATATATGGCC-GAGTGAGCGAGCAGGATCTCCGCTTTGAC-CGCGAAATTTGAACGGCGC"), part of the published sequence for the human collagen intron (NW_927317), to do comparative analysis of the impact of the mutation .
- 86 **DNA fragments:** The propensity of cyclization determines the intrinsic bendability of a DNA fragment which may influence a myriad number of essential cellular mechanisms. Previously Alexandrov *et. al* Alexandrov et al. (2016) studied the impact of cyclization rates based on DNA structural parameters

bioRxiv preprint doi: https://doi.org/10.1101/2023.09.09.557010; this version posted September 12, 2023. The copyright holder for this preprint (which was not certified by peer review) is the author/funder. This article is a US Government work. It is not subject to copyright under 17 USC 105 and is also made available for use under a CC0 license.

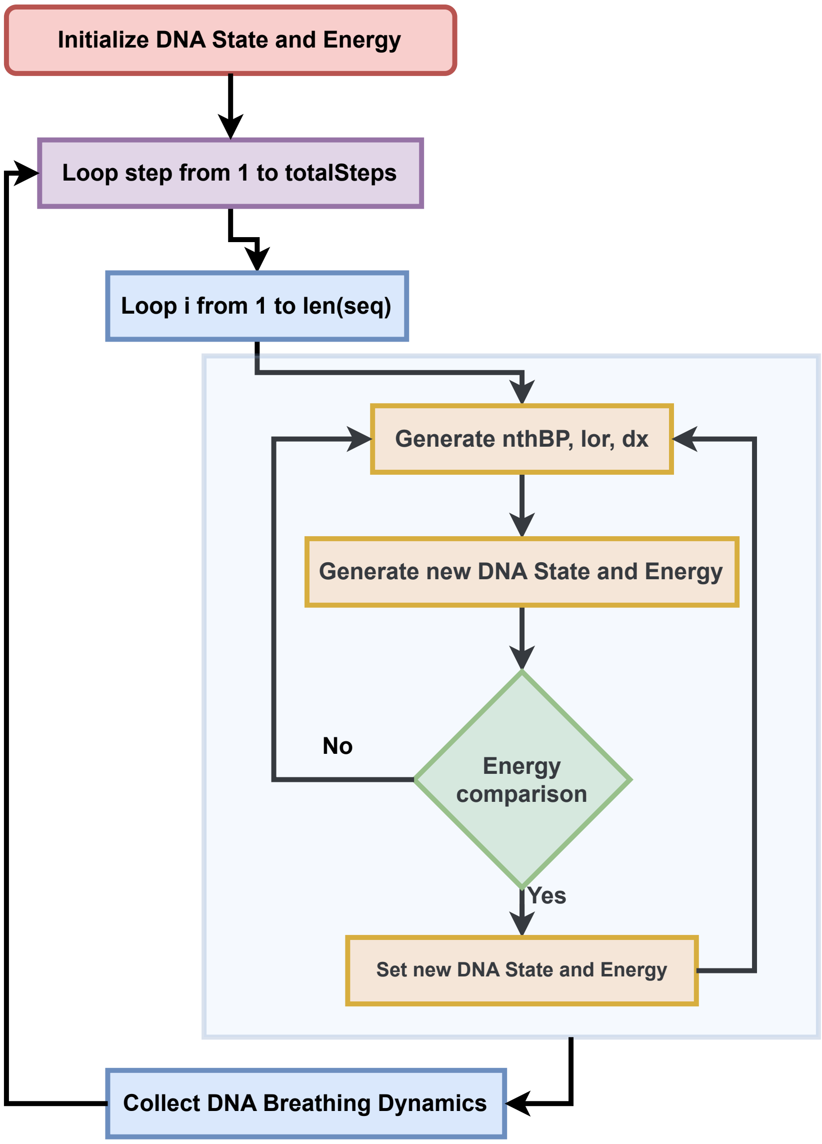

Fig. 2: Schematic representation of the Metropolis-Hasting MCMC Algorithm for DNA State Transitions in pyDNA-EPBD

of 86 DNA fragments of length range from 57 to 325 bp (excluding flank sequence "GC" on both sides in our study) with experimentally determined cyclization factors.

- **Seven SNPs:** To study the functional impact of single nucleotide polymorphisms (SNPs), we studied seven SNPs with the original sequence and allele modified sequence at and around 100 bp of the mutation site. The studied reference SNP ids (rs-ids) are reported in Section B.6.

B.2 pyDNA-EPBD simulation

Given an input DNA sequence, following Algorithm 1, we run at least 100 MCMC simulations with different initial conditions. The temperature is set to 310.0 Kelvin with $50,000$ and $80,000$ preheating and post preheating steps, respectively, throughout the studies. Each random displacement is performed by selecting a random base-pair's updated coordinate cutoff threshold of 25 Å. The breathing dynamics are recorded throughout the post preheating steps at different runs as the samples of the generated Markov chain. The distribution of the samples is computed over the independent runs and normalized over the number of post preheating

bioRxiv preprint doi: https://doi.org/10.1101/2023.09.09.557010; this version posted September 12, 2023. The copyright holder for this preprint (which was not certified by peer review) is the author/funder. This article is a US Government work. It is not subject to copyright under 17 USC 105 and is also made available for use under a CC0 license.

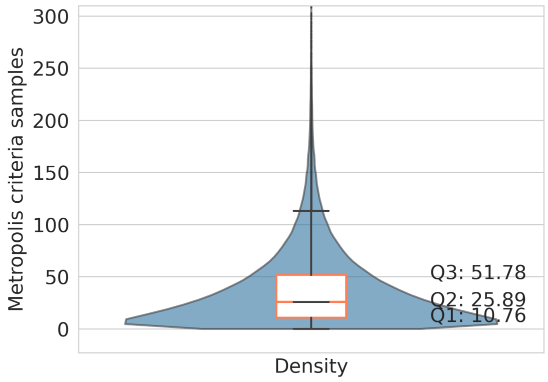

Fig. 3: Continuous probability (Kernel density estimate) distribution of the Metropolis criteria (violin plot, blue area). Y-axis shows the spread of the generated 130,000 samples (simulation temperature scaled in Kelvin) and x-axis gives the density. The box-and-whisker plot facilitates the distribution in quartiles. Both demonstrates pyDNA-EBPD's capability of finding an equilibrium DNA state by exploring its energy landscape.

steps as the final breathing characteristics. The next sections discusses the properties of the simulation outputs as the DNA breathing dynamics in light of capturing several DNA breathing profiles.

When utilizing the pyDNA-EPBD model for analysis of short DNA sequences, the addition of flanking sequences can play a critical role. This is primarily because the biophysical properties of DNA often extend beyond the confines of the sequence of interest and are influenced by the broader bp context. The biophysical traits of a short sequence, in isolation, may not fully encapsulate these sequence-dependent effects. Thus, by including flanking sequences, we are better able to simulate the native context of the DNA sequence, enhancing the accuracy of the model's outputs. Another key factor is the potential distortion of the overall characteristics of short sequences by terminal bps. Given their significant proportion in relation to the total length of the sequence, the terminal bps can have a disproportionate influence on the sequence's characteristics. Therefore, by integrating flanking sequences, we can moderate these boundary effects, ensuring a more representative portrayal of the internal bps within our sequence of interest. Moreover, the pyDNA-EPBD model imposes algorithmic requirements that specify a minimum sequence length. Failure to meet this threshold can lead to inaccurate results. Here, flanking sequences serve a crucial role by augmenting the sequence length to satisfy the model's prerequisites, thereby ensuring the production of reliable features. However, the necessity for flanking sequences diminishes as the length of the sequence increases. Long sequences are typically sufficient to fulfill the model's computational requirements, and the influence of the terminal bps on the overall sequence characteristics becomes negligible, thereby reducing the need for additional flanking sequences.

B.3 Breathing dynamics: Average coordinate distance profile

Alexandrov *et. al* Alexandrov et al. (2009) revealed that the simulation distribution matches the dramatic difference in the transcriptional activity of the promoters. An example showcase is demonstrated for AAV P5 wild- and mutant-promoter sequences. Following the same analysis, we compute the average coordinate distance profile (y_n) for the AAV P5 promoters using our pyDNA-EPBD model. Figure 4 (left panel) shows the average displacements at-and-around bp 50 of the AAV P5 promoter (blue) and a transcriptionally silent AG to TC mutant (red). This result is visually identical as previously reported in Alexandrov et al. (2009). One can utilize the average displacement magnitude in the double helix width as a functional effect on the transcription-factor binding affinity. Moreover, we report the average coordinate profile for 86 different DNA sequences ranging from 61 to 329 bp (including the flanks) in link1.

B.4 Breathing dynamics: Average flipping profile

DNA breathing can expose nucleotide base openings for interaction with proteins, which can be favorable depending on the mechanism of TF-DNA binding Nowak-Lovato et al. (2013). These openings effectively correspond to the base flipping at various sites at physiological temperature and pH, where base flipping gives the base-pair mismatches.

We computed the average flipping profile using our pyDNA-EPBD model as the probability of a base-pair being flipped throughout the simulation steps. A base-pair is considered flipped if the coordinate distance of that base-pair is no less than a threshold (in Å). We collected flipping profiles at five different thresholds from 0.707106781186548 Å to 3.53553390593274 Å, inclusive, with step size 0.707106781186548. Note that the floating point precision is crucial for obtaining accurate distribution. Figure 4 (right panel) demonstrates example flipping profiles for AAV P5 wild- and mutant-promoter sequences at threshold 1.414213562373096 Å. It shows that the transcriptionally silent AAV P5 mutant is less prone to bp openings at-and-around mutation position at certain threshold compared with its counter wild P5 promoter. Thus, average flipping profile is subjected to study more profoundly to understand the functional activity of the SNPs. We also provide the average flipping profile for 86 DNA nucleotide sequences at 1.414213562373096 Å threshold, which can be found in link2.

bioRxiv preprint doi: https://doi.org/10.1101/2023.09.09.557010; this version posted September 12, 2023. The copyright holder for this preprint (which was not certified by peer review) is the author/funder. This article is a US Government work. It is not subject to copyright under 17 USC 105 and is also made available for use under a CC0 license.

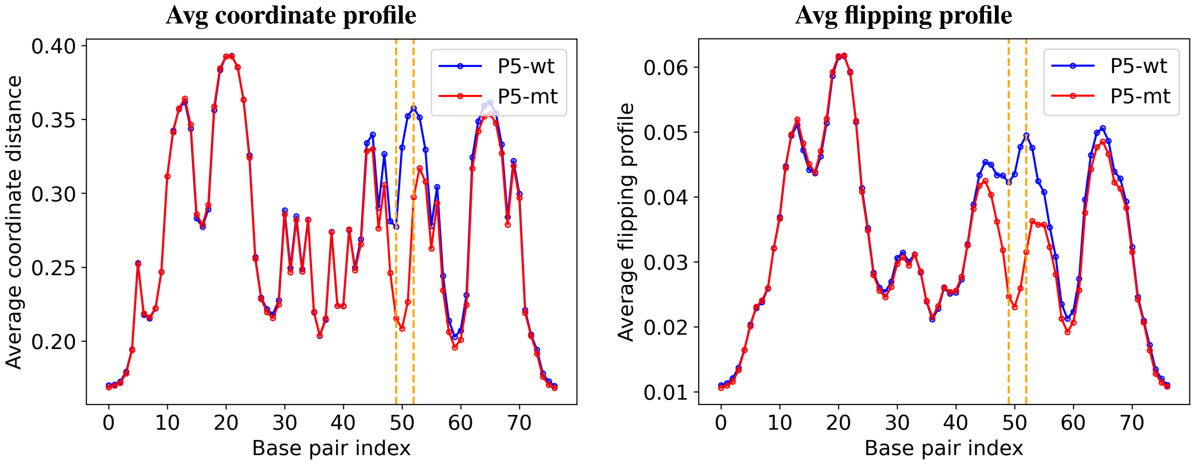

Fig. 4: Average coordinate distance (left) and flipping (right) profile for AAV P5 wild- and mutant-promoter sequences at each BP. The vertical block (orange) shows the mutation of a nucleotide bps substitutions from AG to TC at 50 and 51 positions (zero-indexed).

B.5 Breathing dynamics: Bubbles

We collected bubbles using $P_n(l, thr) = \langle \frac{1}{t_s} \sum_{q_n^k=1}^{q_n^{kmax}} \Delta t[q_n^k(l, thr)] \rangle_M$ from minimum of three bps to maximum of 20 bps incrementally by applying our pyDNA-EPBD simulation tool at 20 different thresholds from 0.5 to 10.0 Å with step size 0.5. Figure 5 demonstrates the bubbles for AAV P5 wild- and mutant-promoter sequences on the left and right panel, respectively. Each row compares the bubbles with its counterparts at a certain threshold.

In the first row, we can observe overall peak distribution when a small bubble detection coordinate distance threshold (thr = 2.5 Å) is considered. As the threshold got increased, bubbles are created at very different locations in AAV P5 wild- and mutant-promoters. This confirms previous findings in Choi (2004); Alexandrov et al. (2006, 2009) that a dramatic difference in the bubble probability at the mutation site in those two sequences has been occurred, matching the dramatic difference in the transcriptional activity of the promoters. Another important observation is that bubbles are only stretched over small number of nucleotide bps, which provides evidence of spontaneity of opening and re-closing of the double stranded DNA. Furthermore, this phenomena has previously been observed by Peyrard Peyrard (2004) in the early work of analyzing dynamical properties in DNA where only the bubbles with small stretching length are found when the simulation temperature is set to less than the denaturation temperature. Since we study all the simulations in this work at 310.0 Kelvin, we only observe small breathing window. One can also observe bubble denaturation using thermal bath by increasing simulation temperature.

B.6 Breathing dynamics: Q-factor

Sequence-dependent DNA breathing dynamics from the nonlinear EPBD model Alexandrov et al. (2009) has been successfully used to demonstrate the effect of SNPs on TF binding via DNA breathing. While the breathing profile or flipping profile represents the average probability of DNA openings at each bp in the region, the MCMC simulation induces statistical errors in the distribution of the breathing characteristics. In Figure 6 (left panel), we show the average flipping profile with 95% confidence interval (shaded region). Thus, here we propose a novel dynamical feature named q-factor to compare the statistical differences in the breathing distribution at each bp between a SNP (wild and mutant pair). First we collect the flipping profile using our pyDNA-EPBD model for a SNP. At each bp we quantify a distance between two Markov distributions of the original sequence and allele modified sequence. We compute a statistical test known as Kolmogorov Smirinov2 test and scale the distances into negative logarithm (base 10). In Figure 6 (right panel), we report the q-factor for AAV P5 wild- and mutant-promoter. Clearly, the q-factor at the mutation gives a functional effect measurement which was not previously readily available.

To summarize the effect of a SNP on DNA breathing in the 100 base-pair region, we computed the q-factor scores of three tested SNPs of PIQ-predicted TF footprints. These SNPs likely affect TF binding and have higher scores when compared to the four control SNPs that are not adjacent to PIQ-predicted TF footprints. As an example, in Figure 7 rs12946510, known to affect TF binding , significantly affects the DNA breathing of 11 base-pair around the SNP. In contrast, rs72974222, which is not adjacent to a PIQ-predicted TF footprint , weakly affects the DNA breathing of 5 base-pair around the SNP. In Figure 7 (bottom panel), we summarize the q-factor scores at the mutation site for

bioRxiv preprint doi: https://doi.org/10.1101/2023.09.09.557010; this version posted September 12, 2023. The copyright holder for this preprint (which was not certified by peer review) is the author/funder. This article is a US Government work. It is not subject to copyright under 17 USC 105 and is also made available for use under a CC0 license.

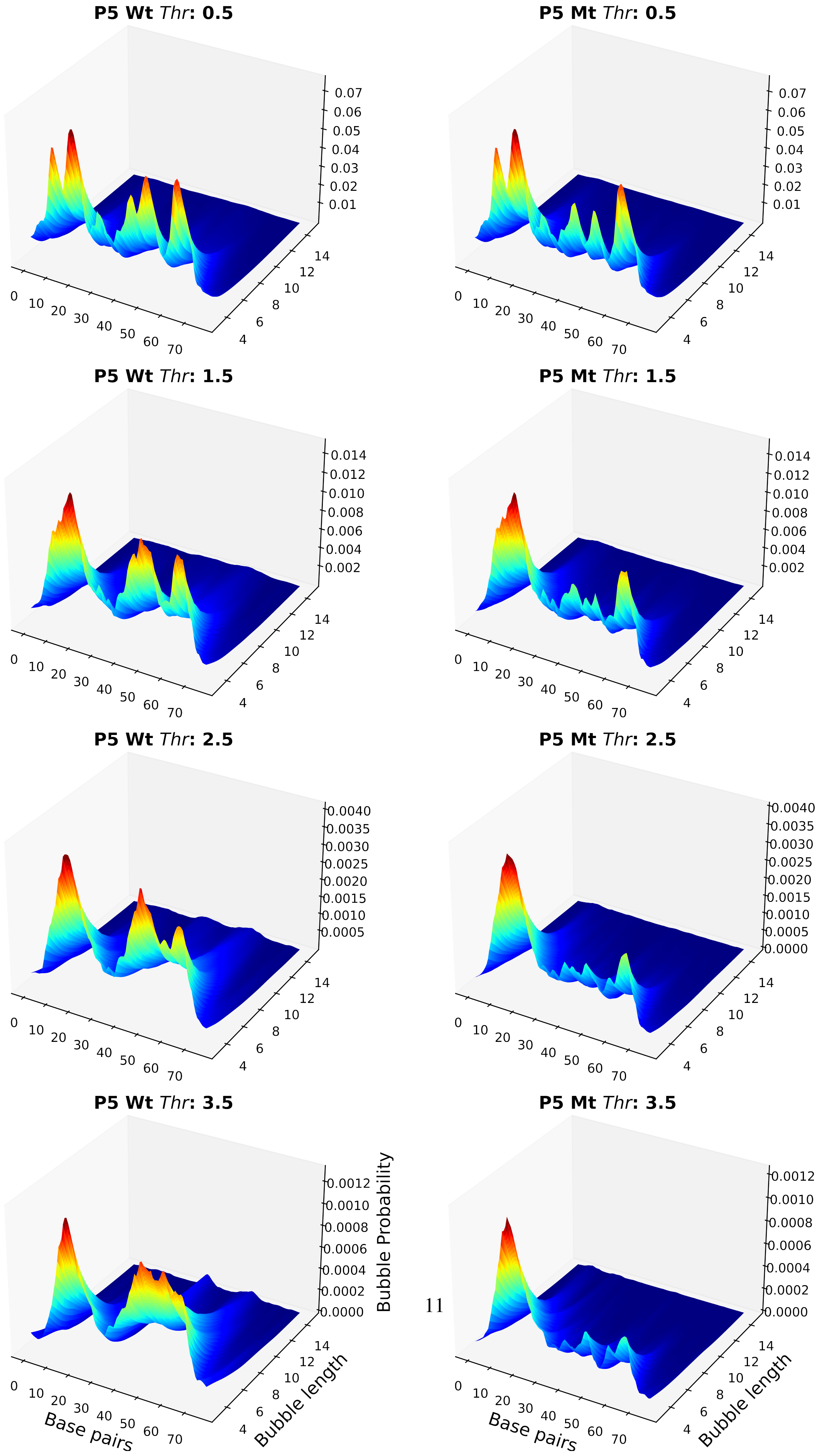

Fig. 5: 3D surface plots representing the change in bubble intensity across various bubble lengths and bps. Each row represents different threshold values for two different conditions: P5 wild (left panel) and P5 mutant (right panel).

bioRxiv preprint doi: https://doi.org/10.1101/2023.09.09.557010; this version posted September 12, 2023. The copyright holder for this preprint (which was not certified by peer review) is the author/funder. This article is a US Government work. It is not subject to copyright under 17 USC 105 and is also made available for use under a CC0 license.

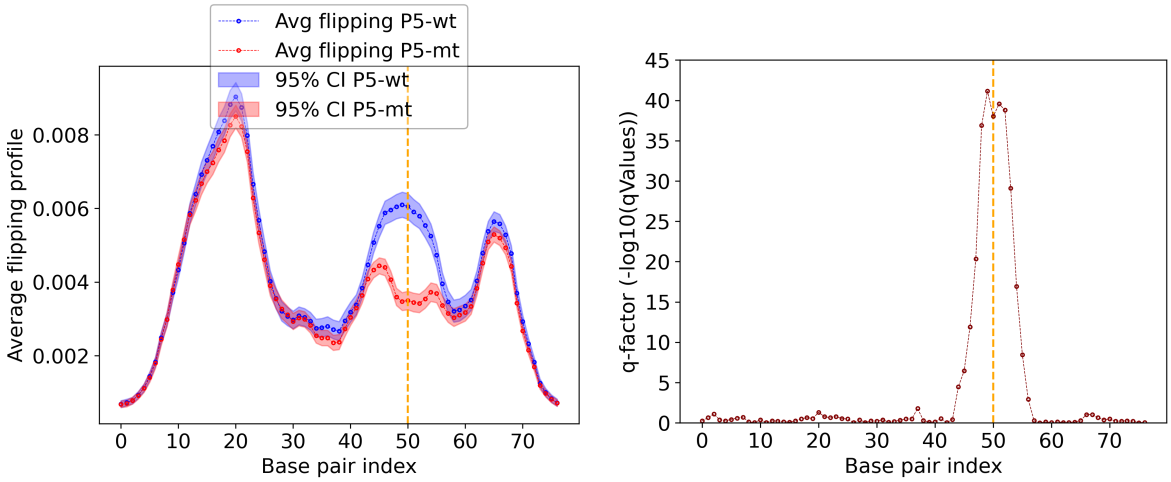

Fig. 6: Average flipping profile with corresponding q-factor for AAV P5 wild- and mutant-sequence.

seven SNPs and P5 promoter. As the figure clearly suggests, the boundary between PIQ-predicted TF footprint and controls SNPs is easily distinguishable. However, it requires an extensive study to draw a cutoff line which we deem as one of the future works.

References

Turton, D.A., Senn, H.M., Harwood, T., Lapthorn, A.J., Ellis, E.M., Wynne, K.: Terahertz underdamped vibrational motion governs protein-ligand binding in solution. Nature communications **5**(1), 3999 (2014)

Guéron, M., Kochoyan, M., Leroy, J.-L.: A single mode of dna base-pair opening drives imino proton exchange. Nature **328**(6125), 89–92 (1987)

Klimašauskas, S., Roberts, R.J.: M hha l binds tightly to substrates containing mismatches at the target base. Nucleic acids research **23**(8), 1388–1395 (1995)

Roberts, R.J., Cheng, X.: Base flipping. Annual review of biochemistry **67**(1), 181–198 (1998)

Choi, C.H., Kalosakas, G., Rasmussen, K.Ø., Hiromura, M., Bishop, A.R., Usheva, A.: Dna dynamically directs its own transcription initiation. Nucleic Acids Research **32**(4), 1584–1590 (2004)

Dellarole, M., Sánchez, I.E., Prat Gay, G.: Thermodynamics of cooperative dna recognition at a replication origin and transcription regulatory site. Biochemistry **49**(48), 10277–10286 (2010)

Fenwick, R.B., Esteban-Martín, S., Salvatella, X.: Understanding biomolecular motion, recognition, and allostery by use of conformational ensembles. European Biophysics Journal **40**, 1339–1355 (2011)

Alexandrov, B.S., Fukuyo, Y., Lange, M., Horikoshi, N., Gelev, V., Rasmussen, K.Ø., Bishop, A.R., Usheva, A.: Dna breathing dynamics distinguish binding from nonbinding consensus sites for transcription factor yy1 in cells. Nucleic acids research **40**(20), 10116–10123 (2012)

Phelps, C., Lee, W., Jose, D., Hippel, P.H., Marcus, A.H.: Single-molecule FRET and linear dichroism studies of DNA breathing and helicase binding at replication fork junctions. Proceedings of the National Academy of Sciences **110**(43), 17320–17325 (2013) https://doi.org/10.1073/pnas.1314862110

Alexandrov, B.S., Valtchinov, V.I., Alexandrov, L.B., Gelev, V., Dagon, Y., Bock, J., Kohane, I.S., Rasmussen, K.Ø., Bishop, A.R., Usheva, A.: Dna dynamics is likely to be a factor in the genomic nucleotide repeats expansions related to diseases. PloS one **6**(5), 19800 (2011)

Jablensky, A., Angelicheva, D., Donohoe, G., Cruickshank, M., Azmanov, D., Morris, D., McRae, A., Weickert, C., Carter, K., Chandler, D., *et al.*: Promoter polymorphisms in two overlapping 6p25 genes implicate mitochondrial proteins in cognitive deficit in schizophrenia. Molecular psychiatry **17**(12), 1328–1339 (2012)

Duan, J., Shi, J., Fiorentino, A., Leites, C., Chen, X., Moy, W., Chen, J., Alexandrov, B.S., Usheva, A., He, D., *et al.*: A rare functional noncoding variant at the gwas-implicated mir137/mir2682 locus might confer risk to schizophrenia and bipolar disorder. The American Journal of Human Genetics **95**(6), 744–753 (2014)

Ares, S., Voulgarakis, N., Rasmussen, K., Bishop, A.R.: Bubble nucleation and cooperativity in dna melting. Physical review letters **94**(3), 035504 (2005)

Alexandrov, B.S., Gelev, V., Monisova, Y., Alexandrov, L.B., Bishop, A.R., Rasmussen, K.Ø., Usheva, A.: A nonlinear dynamic model of dna with a sequence-dependent stacking term. Nucleic Acids Research **37**(7), 2405–2410 (2009)

Alexandrov, B.S., Gelev, V., Yoo, S.W., Alexandrov, L.B., Fukuyo, Y., Bishop, A.R., Rasmussen, K.Ø., Usheva, A.: Dna dynamics play a role as a basal transcription factor in the positioning and

bioRxiv preprint doi: https://doi.org/10.1101/2023.09.09.557010; this version posted September 12, 2023. The copyright holder for this preprint (which was not certified by peer review) is the author/funder. This article is a US Government work. It is not subject to copyright under 17 USC 105 and is also made available for use under a CC0 license.

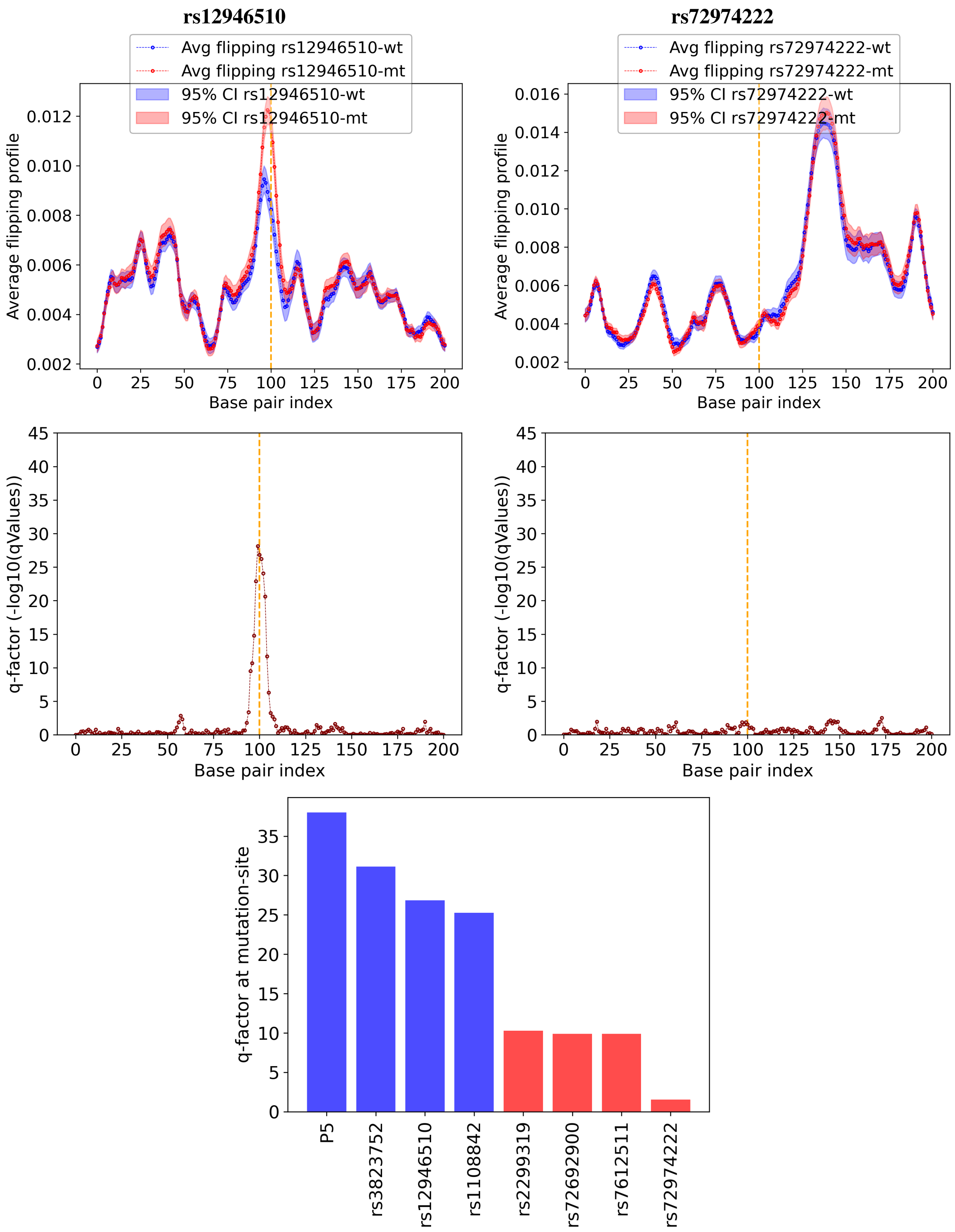

Fig. 7: Average flipping profile with the corresponding q-factor for P5 two SNPs (rs12946510 and rs72974222 on the left and right panel). On the bottom panel, q-factor values for P5 and three PIQ-predicted TF footprint and four controls SNPs.

bioRxiv preprint doi: https://doi.org/10.1101/2023.09.09.557010; this version posted September 12, 2023. The copyright holder for this preprint (which was not certified by peer review) is the author/funder. This article is a US Government work. It is not subject to copyright under 17 USC 105 and is also made available for use under a CC0 license.

regulation of gene transcription initiation. Nucleic acids research **38**(6), 1790–1795 (2010)

Alexandrov, B.S., Gelev, V., Yoo, S.W., Bishop, A.R., Rasmussen, K.Ø., Usheva, A.: Toward a detailed description of the thermally induced dynamics of the core promoter. PLoS Computational Biology **5**(3), 1000313 (2009) https://doi.org/10.1371/journal.pcbi.1000313

Nowak-Lovato, K., Alexandrov, L.B., Banisadr, A., Bauer, A.L., Bishop, A.R., Usheva, A., Mu, F., Hong-Geller, E., Rasmussen, K.Ø., Hlavacek, W.S., *et al.*: Binding of nucleoid-associated protein fis to dna is regulated by dna breathing dynamics. PLoS Computational Biology **9**(1), 1002881 (2013)

Blagoev, K., Alexandrov, B., Goodwin, E., Bishop, A.: Ultra-violet light induced changes in dna dynamics may enhance tt-dimer recognition. DNA repair **5**(7), 863–867 (2006)

Bock, J., Fukuyo, Y., Kang, S., Phipps, M.L., Alexandrov, L.B., Rasmussen, K.Ø., Bishop, A.R., Rosen, E.D., Martinez, J.S., Chen, H.-T., *et al.*: Mammalian stem cells reprogramming in response to terahertz radiation. PloS one **5**(12), 15806 (2010)

Alexandrov, B.S., Rasmussen, K.Ø., Bishop, A.R., Usheva, A., Alexandrov, L.B., Chong, S., Dagon, Y., Booshehri, L.G., Mielke, C.H., Phipps, M.L., *et al.*: Non-thermal effects of terahertz radiation on gene expression in mouse stem cells. Biomedical Optics Express **2**(9), 2679–2689 (2011)

Alexandrov, B.S., Phipps, M.L., Alexandrov, L.B., Booshehri, L.G., Erat, A., Zabolotny, J., Mielke, C.H., Chen, H.-T., Rodriguez, G., Rasmussen, K.Ø., *et al.*: Specificity and heterogeneity of terahertz radiation effect on gene expression in mouse mesenchymal stem cells. Scientific reports **3**(1), 1184 (2013)

Titova, L.V., Ayesheshim, A.K., Golubov, A., Rodriguez-Juarez, R., Kovalchuk, A., Hegmann, F.A., Kovalchuk, O.: Intense picosecond thz pulses alter gene expression in human skin tissue in vivo. In: Terahertz and Ultrashort Electromagnetic Pulses for Biomedical Applications, vol. 8585, pp. 117–126 (2013). SPIE

Perera, P.G.T., Appadoo, D.R., Cheeseman, S., Wandiyanto, J.V., Linklater, D., Dekiwadia, C., Truong, V.K., Tobin, M.J., Vongsvivut, J., Bazaka, O., *et al.*: Pc 12 pheochromocytoma cell response to super high frequency terahertz radiation from synchrotron source. Cancers **11**(2), 162 (2019)

Shang, S., Wu, X., Zhang, Q., Zhao, J., Hu, E., Wang, L., Lu, X.: 0.1 thz exposure affects primary hippocampus neuron gene expression via alternating transcription factor binding. Biomedical Optics Express **12**(6), 3729–3742 (2021)

González-Jiménez, M., Ramakrishnan, G., Harwood, T., Lapthorn, A.J., Kelly, S.M., Ellis, E.M., Wynne, K.: Observation of coherent delocalized phonon-like modes in dna under physiological conditions. Nature communications **7**(1), 11799 (2016)

Vafabakhsh, R., Ha, T.: Extreme bendability of dna less than 100 base pairs long revealed by single-molecule cyclization. Science **337**(6098), 1097–1101 (2012)

Alexandrov, L.B., Bishop, A.R., Rasmussen, K.Ø., Alexandrov, B.S.: The role of structural parameters in dna cyclization. BMC bioinformatics **17**(1), 1–10 (2016)

Yan, J., Marko, J.F.: Localized single-stranded bubble mechanism for cyclization of short double helix dna. Physical review letters **93**(10), 108108 (2004)

Alexandrov, L.B., Rasmussen, K.Ø., Bishop, A.R., Alexandrov, B.S.: Evaluating the role of coherent delocalized phonon-like modes in dna cyclization. Scientific reports **7**(1), 9731 (2017)

Choi, C.H.: DNA dynamically directs its own transcription initiation. Nucleic Acids Research **32**(4), 1584–1590 (2004) https://doi.org/10.1093/nar/gkh335

Alexandrov, B., Wille, L., Rasmussen, K., Bishop, A., Blagoev, K.: Bubble statistics and dynamics in double-stranded dna. Physical review E **74**(5), 050901 (2006)

Lai, Y.: A statistical method for the conservative adjustment of false discovery rate (q-value). BMC bioinformatics **18**(3), 155–163 (2017)

Dauxois, T., Peyrard, M., Bishop, A.R.: Entropy-driven DNA denaturation. Physical Review E **47**(1), 44–47 (1993) https://doi.org/10.1103/physreve.47.r44

Richard, C., Guttmann, A.J.: Poland–scheraga models and the DNA denaturation transition. Journal of Statistical Physics **115**(3/4), 925–947 (2004) https://doi.org/10.1023/b:joss.0000022370.48118.8b

Alexandrov, L.B., Bishop, A.R., Rasmussen, K.Ø., Alexandrov, B.S.: The role of structural parameters in DNA cyclization. BMC Bioinformatics **17**(1) (2016) https://doi.org/10.1186/s12859-016-0897-9

Nowak-Lovato, K., Alexandrov, L.B., Banisadr, A., Bauer, A.L., Bishop, A.R., Usheva, A., Mu, F., Hong-Geller, E., Rasmussen, K.Ø., Hlavacek, W.S., Alexandrov, B.S.: Binding of nucleoid-associated protein fis to DNA is regulated by DNA breathing dynamics. PLoS Computational Biology **9**(1), 1002881 (2013) https://doi.org/10.1371/journal.pcbi.1002881

Alexandrov, B.S., Wille, L.T., Rasmussen, K.Ø., Bishop, A.R., Blagoev, K.B.: Bubble statistics and dynamics in double-stranded DNA. Physical Review E **74**(5) (2006) https://doi.org/10.1103/physreve.74.050901

Peyrard, M.: Nonlinear dynamics and statistical physics of DNA. Nonlinearity **17**(2), 1–40 (2004) https://doi.org/10.1088/0951-7715/17/2/r01
